# Factors for incidence risk and prognosis of synchronous brain metastases in pulmonary large cell carcinoma patients: a population-based study

**DOI:** 10.1186/s12890-023-02312-y

**Published:** 2023-01-12

**Authors:** Xuan Zheng, Shuai Mu, Lijie Wang, Haitao Tao, Di Huang, Ziwei Huang, Xiaoyan Li, Pengfei Cui, Tao Li, Qingyan Liu, Yi Hu

**Affiliations:** 1 Medical School of Chinese PLA, Beijing, China; 2 Department of Oncology, The First Medical Center of PLA General Hospital, Beijing, China

**Keywords:** Large cell carcinoma, Synchronous brain metastases, Prognosis, Risk factors, SEER

## Abstract

**Background:**

Patients with pulmonary large cell carcinoma (LCC) have a high incidence of synchronous brain metastases (SBM) and a poor prognosis. Our study was to evaluate the predictive and prognostic value of the clinical characteristics of pulmonary LCC patients with SBM at initial diagnosis by utilizing the Surveillance, Epidemiology, and End Results (SEER) database.

**Methods:**

LCC patients, diagnosed from 2010 to 2019, were identified from the latest SEER database which was released in April 2022. Logistic regression and Cox regression were used to identify the predictive and prognostic factors for LCC patients with SBM. Propensity score matching (PSM) and Kaplan–Meier analyses were applied to assess different therapy modalities.

**Results:**

A total of 1375 LCC patients were enrolled in this study and 216 (15.7%) of them had SBM at the initial diagnosis. The median overall survival (OS) of LCC patients with SBM was 4 months. Multivariate Cox regression identified age 60–79 (OR 0.57; 95% CI 0.41–0.78; *p* < 0.001), age ≥ 80 (OR 0.23; 95% CI 0.12–0.45; *p* < 0.001) and bone metastases (OR 1.75; 95% CI 1.22–2.51; *p* < 0.001) as significant independent predictors for developing SBM. Multivariable Cox regression revealed that age 60–79, T stage, bone metastases and chemotherapy were independent prognostic factor for OS. The surgery combined with chemotherapy and radiotherapy group, in which all patients were N0 stage and had no other site-specific metastases, exhibited the best median OS of 15 months.

**Conclusions:**

LCC patients with age < 60 or bone metastases were more likely to have SBM at initial diagnosis. Age, T stage, bone metastases and chemotherapy were independent prognostic factors for OS of LCC patients with SBM. Highly selected patients might achieve the best survival benefit from surgery combined with chemotherapy and radiotherapy.

**Supplementary Information:**

The online version contains supplementary material available at 10.1186/s12890-023-02312-y.

## Background

Large cell carcinoma (LCC) is a rare subtype of non-small cell lung cancer (NSCLC), accounting for just 1.3% of all cases of lung cancer [1]. According to the WHO classification criteria, LCC is an undifferentiated tumor, which lacks histological features and immunomarkers for neuroendocrine, squamous, or glandular differentiation [2]. LCC possesses strong invasive and proliferative characteristics [3]. Currently, there is no recommended treatment for LCC, thus its treatments are often decided according to guidelines of other types of NSCLC. However, due to lack of targeted therapies and specific molecular markers, the prognosis of LCC is poor [4]. Previous study has reported that approximately 48% of all NSCLC patients exhibited distant metastasis at diagnosis, and brain metastases is the most common single metastatic site for patients with LCC [5]. Brain metastases, leading to neurologic symptoms and functional and emotional impairment, are associated with significant morbidity and poor survival outcomes [6]. In NSCLC patients with synchronous brain metastases (SBM) at initial diagnosis, the 1-year OS rate was 22.1%, which was much lower than that in NSCLC patients without SBM [7]. With the development of targeted therapy and immunotherapy, the overall survival of patients with lung cancer has significantly increased. As a consequence, the incidence of BM also increases up to 50% of patients during the course of their illness [8].

Up to now, due to its rareness, there is still little information about the factors affecting incidence and prognosis of LCC patients with SBM. The Surveillance, Epidemiology, and End Results (SEER) database is a population-based clinical oncology repository maintained by National Cancer Institute. It recorded information about incidence, mortality, and morbidity of confirmed frequent malignancies, covering approximately 34% of the United States population. Therefore, by utilizing the SEER database, we aimed to analyze the risk factors for SBM incidence and prognostic factors of OS in LCC patients with SBM. We also estimated the impact of different therapeutic strategies on OS of LCC patients with SBM.

## Methods

### Data collection

The data analyzed in this study were obtained from Surveillance, Epidemiology, and End Results (SEER) 17 Registries (November 2021 submission). Because SEER did not record BM information until 2010, patients diagnosed before that year were excluded. Inclusion criteria were as follows: (1) age ≥ 18 years; (2) year of diagnosis from 2010 to 2019; (3) pathologically confirmed LCC based on histology, according to the International Classification of Diseases for Oncology, Third Edition (ICD-O-3), histology code 8012/3 (Large cell carcinoma, NOS); (4) tumor site location in lung and bronchus; (5) only one primary tumor. Histology code 8013/3 (large cell neuroendocrine carcinoma) and 8014/3 (large cell with rhabdoid phenotype) were not included in this study because these two types have been grouped with other types of NSCLC since the 2015 World Health Organization (WHO) Lung Tumors Classification. In addition, we excluded pathological grade I (well differentiated) and grade II (moderately differentiated) that were not in accordance with the pathological requirements of large cell carcinoma. Patients with incomplete demographic or clinical information were also excluded from this study. The following covariates were collected from the database: year of diagnosis, age at diagnosis, sex, race, marital status, median household income, primary site, laterality, grade, derived AJCC T stage, N stage, synchronous tumor metastases, RX Summ-Surg Prim Site, chemotherapy recode, radiation therapy, survival months, vital status recode, and SEER cause-specific death classification. During the preprocessing phase, AJCC T stages were recalculated based on the guidelines of the AJCC Cancer Staging Manual eighth Edition.

### Study design and statistical analysis

Pearson’s chi-square test and Fisher's exact test were used to compare the demographics, clinical features and treatment differences between LCC patients with and without SBM. To identify the risk factors of SBM incidence, univariate and multivariate logistic regression analyses were performed. Overall survival (OS) was defined as the time from the date of diagnosis to the date of death, or patients who were reported to be alive at the last follow-up date. Because only 5 patients with SBM (2.3%) died of causes other than cancer, lung cancer-specific survival (LCSS) was not estimated in this study. The OS curves were calculated using the Kaplan–Meier method and differences between groups were compared by log-rank test. Univariate and multivariate Cox regression analyses were conducted to determine the independently prognostic factors affecting OS. In the process of comparing the effects of different therapeutic strategies on patients with SBM, surgery alone and surgery combined with radiotherapy were removed from this part due to a quite small proportion (1 patient and 3 patients respectively). We used the propensity score matching (PSM) method to further compare the differences in survival between the chemotherapy and radiotherapy group and the chemotherapy alone group, as well as between the radiotherapy alone group and the no treatment group. All P values were two-sided and *p* < 0.05 was considered to indicate a statistically significant difference. Statistical analysis was performed using R version 4.1.1 software (http://www.r-project.org/).

## Results

### Patients characteristics

**Fig. 1:**
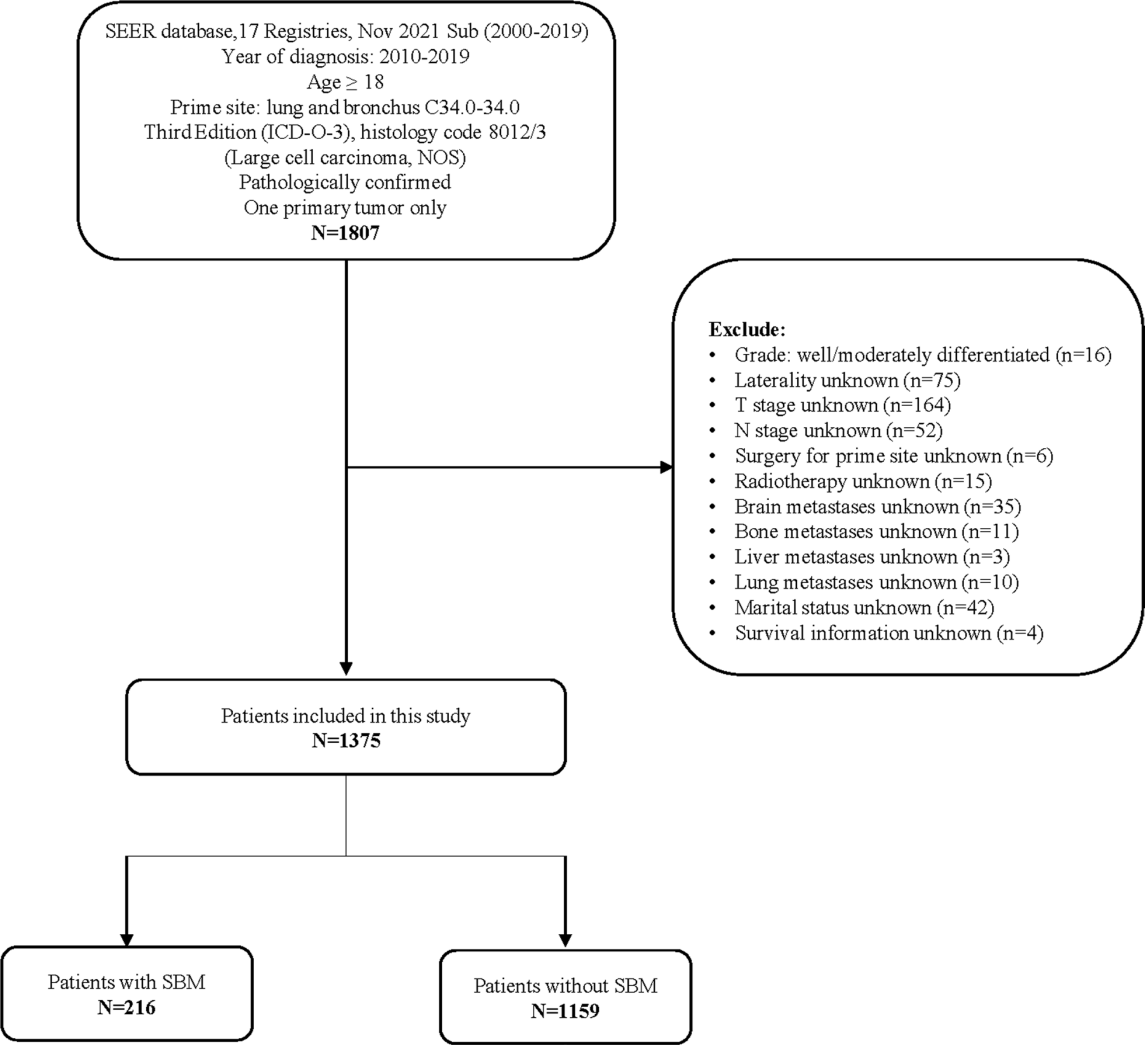
The flowchart of patient selection in this study. SBM, synchronous brain metastases

A total of 1375 LCC patients diagnosed from 2010 to 2019 were enrolled in this study and 216 (15.7%) of them had SBM at the initial diagnosis (Fig. 1). Table 1 shows the demographic and clinical characteristics of all patients. The number of patients diagnosed as LCC between 2015 and 2019 (n = 419) decreased by more than half compared with 2010–2014 (n = 956). The majority of LCC patients (73.2%) are older than 60 years old, while the proportion of young patients (age 25–59) in the SBM group was higher than the non-SBM group (38.4% vs 24.6%). Male patients (n = 826) were more than female patients (n = 549), but no significant gender predominance was found in the presence of brain metastases. In terms of clinical characteristics, the SBM group had higher rate of T3 and T4 stage (26.4% vs 21.9, 41.2% vs 38.8%), but that difference was not statistically significant (*p* = 0.327). The SBM group had significantly higher rate of N2 stage (46.8% vs 39.6%), bone metastases (33.3% vs 18.4%), liver metastases (18.5% vs 9.4%), lung metastasis (19.9% vs 13.3%).

**Table 1:** Baseline characteristics between LCC patients with and without SBM

Characteristics	Overall	With SBM	Without SBM	*p* Value
	N = 1375	N = 216	N = 1159	
*Year of diagnosis*				0.239
2010–2014	956 (69.53)	158 (73.15)	798 (68.85)	
2015–2019	419 (30.47)	58 (26.85)	361 (31.15)	
*Age*				
< 60	368 (26.76)	83 (38.43)	285 (24.59)	< 0.001
60–79	841 (61.16)	122 (56.48)	719 (62.04)	
≥ 80	166 (12.07)	11 (5.09)	155 (13.37)	
*Sex*				0.344
Male	826 (60.07)	123 (56.94)	703 (60.66)	
Female	549 (39.93)	93 (43.06)	456 (39.34)	
*Race*				0.811
White	1118 (81.31)	178 (82.41)	940 (81.10)	
Black	188 (13.67)	29 (13.43)	159 (13.72)	
Other	69 (5.02)	9 (4.17)	60 (5.18)	
*Marital status*				0.999
Married	697 (50.69)	110 (50.93)	587 (50.65)	
Single	678 (49.31)	106 (49.07)	572 (49.35)	
*Median household income*				0.293
< $55,000	556 (40.44)	81 (37.50)	475 (40.98)	
$55,000–$74,999	577 (41.96)	101 (46.76)	476 (41.07)	
$75,000+	242 (17.60)	34 (15.74)	208 (17.95)	
*Primary site*				0.892
Main bronchus	61 (4.44)	9 (4.17)	52 (4.49)	
Upper lobe	799 (58.11)	128 (59.26)	671 (57.89)	
Middle lobe	46 (3.35)	5 (2.31)	41 (3.54)	
Lower lobe	314 (22.84)	52 (24.07)	262 (22.61)	
Unspecific	155 (11.27)	22 (10.19)	133 (11.48)	
*Laterality*				0.438
Left	570 (41.45)	97 (44.91)	473 (40.81)	
Right	788 (57.31)	116 (53.70)	672 (57.98)	
Bilateral	17 (1.24)	3 (1.39)	14 (1.21)	
*Grade*				0.044
III	468 (34.04)	67 (31.02)	401 (34.60)	
IV	429 (31.20)	58 (26.85)	371 (32.01)	
Unknown	478 (34.76)	91 (42.13)	387 (33.39)	
*T stage*				0.327
T1	208 (15.13)	25 (11.57)	183 (15.79)	
T2	302 (21.96)	43 (19.91)	259 (22.35)	
T3	310 (22.55)	57 (26.39)	253 (21.83)	
T4	539 (39.20)	89 (41.20)	450 (38.83)	
T0	16 (1.16)	2 (0.93)	14 (1.21)	
*N stage*				0.050
N0	476 (34.62)	57 (26.39)	419 (36.15)	
N1	122 (8.87)	21 (9.72)	101 (8.71)	
N2	560 (40.73)	101 (46.76)	459 (39.60)	
N3	217 (15.78)	37 (17.13)	180 (15.53)	
*Bone metastases*				< 0.001
No	1090 (79.27)	144 (66.67)	946 (81.62)	
Yes	285 (20.73)	72 (33.33)	213 (18.38)	
*Liver metastases*				< 0.001
No	1226 (89.16)	176 (81.48)	1050 (90.60)	
Yes	149 (10.84)	40 (18.52)	109 (9.40)	
*Lung metastases*				0.015
No	1178 (85.67)	173 (80.09)	1005 (86.71)	
Yes	197 (14.33)	43 (19.91)	154 (13.29)	
*Surgery for primary site*				< 0.001
No	1050 (76.36)	206 (95.37)	844 (72.82)	
Yes	325 (23.64)	10 (4.63)	315 (27.18)	
*Chemotherapy*				0.363
No	717 (52.15)	106 (49.07)	611 (52.72)	
Yes	658 (47.85)	110 (50.93)	548 (47.28)	
*Radiation*				< 0.001
No	743 (54.04)	53 (24.54)	690 (59.53)	
Yes	632 (45.96)	163 (75.46)	469 (40.47)	

*LCC* large cell carcinoma, *SBM* Synchronous brain metastasis, *T* Tumor, *N* Node

### Risk factors for SBM incidence

As shown in Table 2, univariate logistic regression analysis revealed age, grade, T stage, N stage, bone metastases, liver metastases, lung metastases as significant predictors for SBM. After adjustment of covariates using multivariate cox regression analysis, age 60–79 (OR 0.57; 95% CI 0.41–0.78; *p* < 0.001) and age ≥ 80 (OR 0.23; 95% CI 0.12–0.45; *p* < 0.001) were identified as independent protective factor for having SBM at diagnosis, while bone metastases (OR 1.75; 95% CI 1.22–2.51; *p* < 0.001) was identified as independent risk factor. A trend for liver metastases towards statistical significance also emerged (OR 1.5; 95% CI 0.96–2.34; *p* = 0.075).

**Table 2:** Univariate and multivariate logistic regression analysis for the risk of SBM at the initial diagnosis of LCC patients

Characteristics	Univariate		Multivariate	
	OR (95% CI)	*p* Value	OR (95% CI)	*p* Value
*Year of diagnosis*				
2010–2014	Reference	–		
2015–2019	0.81 (0.59–1.12)	0.208		
*Age*				
< 60	Reference	–	Reference	–
60–79	0.58 (0.43–0.8)	0.001	0.57 (0.41–0.78)	0.001
≥ 80	0.24 (0.13–0.47)	< 0.001	0.23 (0.12–0.45)	< 0.001
*Sex*				
Male	Reference	–		
Female	1.17 (0.87–1.56)	0.307		
*Race*				
White	Reference	–		
Black	0.96 (0.63–1.48)	0.863		
Other	0.79 (0.39–1.63)	0.525		
*Marital status*				
Married	Reference	–		
Single	0.99 (0.74–1.32)	0.94		
*Median household income*				
< $55,000	Reference	–		
$55,000–$74,999	1.24 (0.9–1.71)	0.179		
$75,000+	0.96 (0.62–1.48)	0.848		
*Primary site*				
Main bronchus	Reference	–		
Upper lobe	1.1 (0.53–2.29)	0.795		
Middle lobe	0.7 (0.22–2.26)	0.557		
Lower lobe	1.15 (0.53–2.47)	0.727		
Unspecific	0.96 (0.41–2.21)	0.916		
*Laterality*				
Left	Reference	–		
Right	0.84 (0.63–1.13)	0.251		
Bilateral	1.04 (0.29–3.71)	0.946		
*Grade*				
III	Reference	–	Reference	–
IV	0.94 (0.64–1.37)	0.731	0.95 (0.64–1.39)	0.778
Unknown	1.41 (1–1.99)	0.052	1.25 (0.88–1.79)	0.219
*T stage*				
T1	Reference	–	Reference	–
T2	1.22 (0.72–2.06)	0.469	1.08 (0.63–1.86)	0.775
T3	1.65 (0.99–2.74)	0.053	1.37 (0.81–2.33)	0.238
T4	1.45 (0.9–2.33)	0.127	1.1 (0.67–1.82)	0.704
T0	1.05 (0.22–4.88)	0.955	0.83 (0.17–3.99)	0.814
*N stage*				
N0	Reference	–	Reference	–
N1	1.53 (0.89–2.64)	0.127	1.4 (0.8–2.46)	0.241
N2	1.62 (1.14–2.3)	0.007	1.29 (0.89–1.88)	0.179
N3	1.51 (0.96–2.37)	0.072	1.17 (0.72–1.88)	0.527
*Bone metastases*				
No	Reference	–	Reference	–
Yes	2.22 (1.61–3.06)	< 0.001	1.75 (1.22–2.51)	0.002
*Liver metastases*				
No	Reference	–	Reference	–
Yes	2.19 (1.47–3.25)	< 0.001	1.5 (0.96–2.34)	0.075
*Lung metastases*				
No	Reference	–	Reference	–
Yes	1.62 (1.12–2.36)	0.011	1.28 (0.84–1.93)	0.249

*LCC* large cell carcinoma, *SBM* Synchronous brain metastasis, *T* Tumor; *N* Node

### Prognosis and survival analysis of LCC patients with SBM

**Fig. 2:**
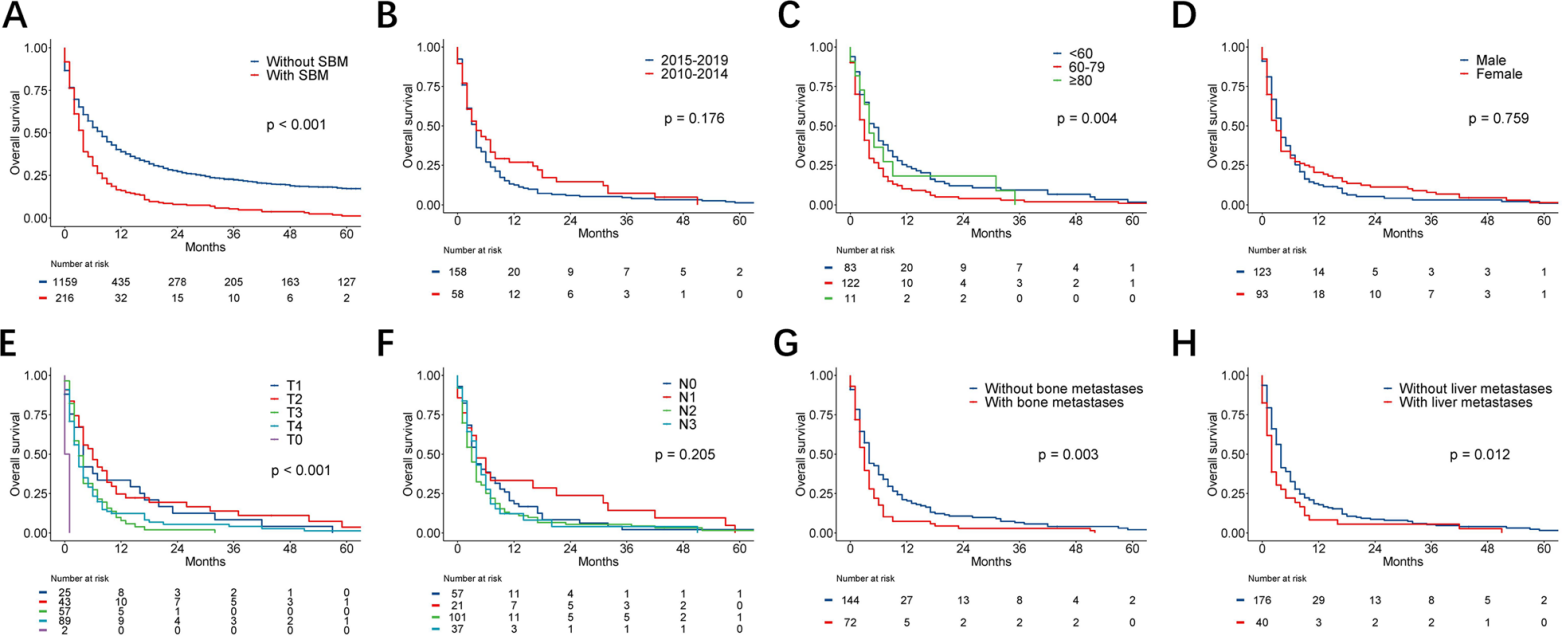
Kaplan–Meier curves of LCC patients based on the presence of synchronous brain metastases. SBM, synchronous brain metastases

94.0% of LCC patients with SBM died at the end of the time-point, and only 5 patients died of causes other than tumors. The median overall survival for the entire cohort was 6 months but the mOS of the SBM group was shorter than those of non-SBM group (4 months vs 8 month, *p* < 0.001, Fig. 2A). The 1-year and 3-years survival rates of patients with SBM were much shorter than those of patients without SBM (30.6% vs. 53.1% and 4.9% vs. 22.4%, respectively, *p* < 0.001). Survival analysis of patients with SBM were further stratified by year of diagnosis (Fig. 2B), age (Fig. 2C), sex (Fig. 2D), T stage (Fig. 2E), N stage (Fig. 2F), bone metastasis (Fig. 2G), liver metastasis (Fig. 2H). The log-rank test showed significant difference of the survival curves between age, T stage, N stage, liver metastases, and bone metastases. Univariate and multivariate cox regression analysis were used to screen the independent prognostic factors for OS in LCC patients with SBM (Table 3). As can be seen in Table 3, the following variables were independent prognostic factors for OS: Age 60–79 (HR = 1.55; 95% CI 1.14–2.10; *p* < 0.001), T stage (T3 vs T1, HR = 2.31; 95% CI 1.36–3.92; *p* < 0.001; T0 vs. T1, HR = 29.95; 95% CI 6.57–136.51; *p* < 0.001), bone metastases (HR = 1.81; 95% CI 1.30–2.50; *p* < 0.001)and chemotherapy (HR = 0.26; 95% CI 0.19–0.36; p < 0.001). A trend for T4 stage (HR = 1.62; 95% CI 0.99–2.65; *p* = 0.056) towards significantly independent prognostic factor also emerged.

**Table 3:** Prognostic factors for median overall survival in LCC patients with SBM

Characteristics	Univariate		Multivariate	
	HR (95% CI)	*p* Value	HR (95% CI)	*p* Value
*Year of diagnosis*				
2010–2014	Reference	–		
2015–2019	0.8 (0.58–1.1)	0.168		
*Age*				
< 60	Reference	–	Reference	–
60–79	1.63 (1.22–2.19)	0.001	1.55 (1.14, 2.10)	0.005
≥ 80	1.17 (0.62–2.21)	0.622	0.71 (0.36, 1.38)	0.311
*Sex*				
Male	Reference	–		
Female	0.97 (0.73–1.28)	0.816		
*Race*				
White	Reference	–		
Black	0.75 (0.5–1.13)	0.174		
Other	0.63 (0.32–1.24)	0.183		
*Marital status*				
Married	Reference	–		
Single	0.97 (0.73–1.28)	0.833		
*Median household income*				
< $55,000	Reference	–		
$55,000–$74,999	0.94 (0.69–1.27)	0.676		
$75,000+	0.93 (0.6–1.44)	0.736		
*Primary site*				
Main bronchus	Reference	–		
Upper lobe	0.83 (0.42–1.64)	0.596		
Middle lobe	1.04 (0.35–3.11)	0.943		
Lower lobe	1.05 (0.52–2.15)	0.885		
Unspecific	0.89 (0.4–1.96)	0.765		
*Laterality*				
Left	Reference	–		
Right	0.97 (0.74–1.29)	0.855		
Bilateral	2.1 (0.66–6.69)	0.207		
*Grade*				
III	Reference	–		
IV	0.9 (0.62–1.3)	0.562		
Unknown	1 (0.72–1.4)	0.986		
*T stage*				
T1	Reference	–	Reference	–
T2	0.84 (0.5–1.39)	0.495	0.99 (0.58, 1.69)	0.973
T3	1.52 (0.93–2.48)	0.095	2.31 (1.36, 3.92)	0.002
T4	1.46 (0.93–2.31)	0.103	1.62 (0.99, 2.65)	0.056
T0	12.18 (2.78–53.36)	0.001	29.95 (6.57, 136.51)	< 0.001
*N stage*				
N0	Reference	–		
N1	0.81 (0.49–1.35)	0.415		
N2	1.28 (0.92–1.8)	0.145		
N3	1.19 (0.77–1.83)	0.442		
*Bone metastases*				
No	Reference	–	Reference	–
Yes	1.57 (1.17–2.11)	0.003	1.81 (1.30, 2.50)	< 0.001
*Liver metastases*				
No	Reference	–	Reference	–
Yes	1.57 (1.1–2.23)	0.013	1.06 (0.73, 1.55)	0.743
*Lung metastases*				
No	Reference	–		
Yes	1.07 (0.75–1.53)	0.718		
*Surgery for primary site*				
No	Reference		Reference	–
Yes	0.57 (0.29–1.11)	0.1	0.55 (0.27, 1.12)	0.099
*Chemotherapy*				
No	Reference		Reference	–
Yes	0.31 (0.23–0.41)	< 0.001	0.26 (0.19, 0.36)	< 0.001
*Radiation*				
No	Reference		Reference	–
Yes	0.56 (0.4–0.78)	0.001	0.78 (0.54, 1.11)	0.166

*LCC* large cell carcinoma, *SBM* Synchronous brain metastasis, *T* Tumor, *N* Node

### Impact of therapy on OS of patients with SBM

**Fig. 3:**
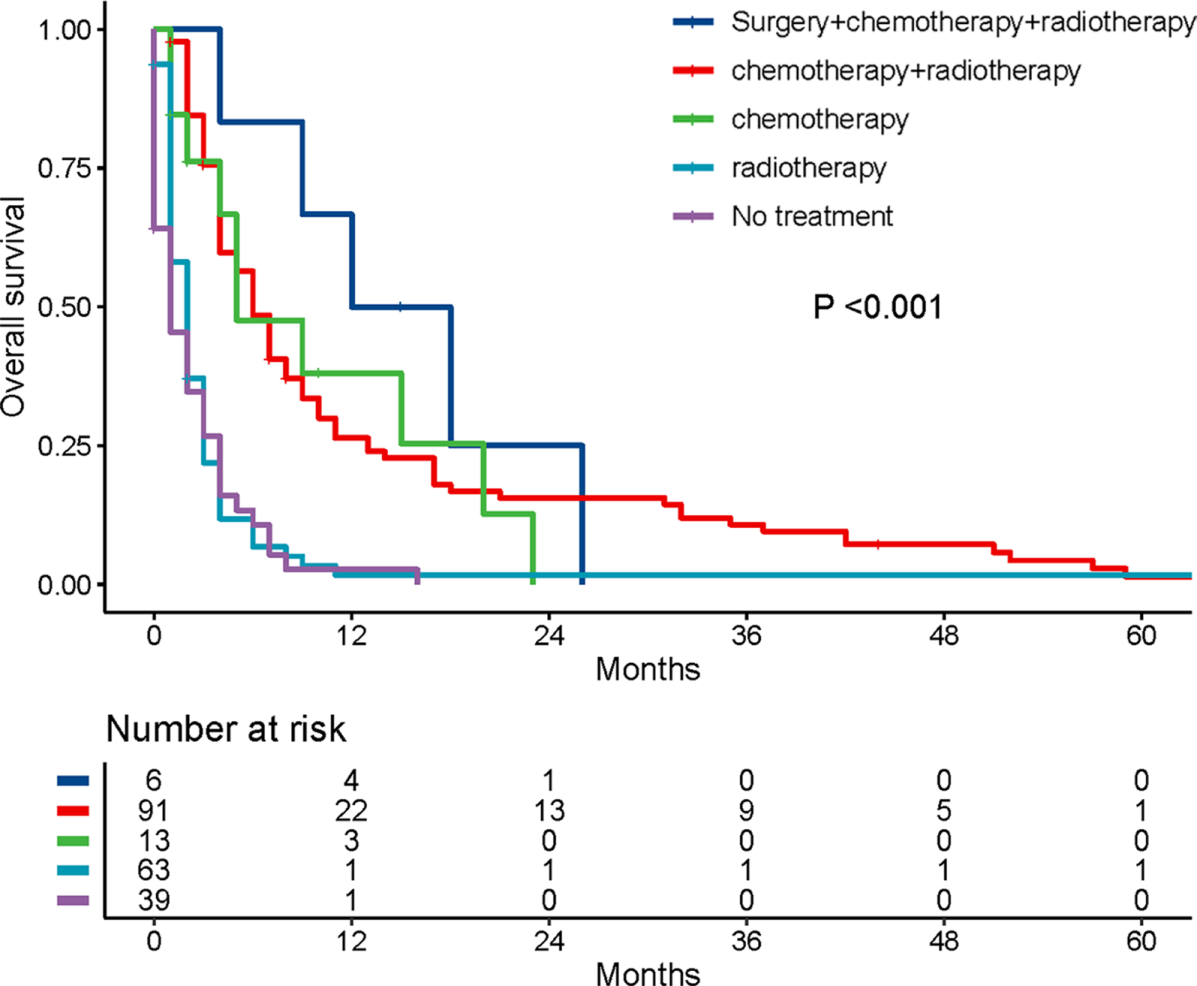
Kaplan–Meier curves of LCC patients with SBM receiving different treatment regimens. LCC, large cell carcinoma; SBM, synchronous brain metastases

47.9% of all LCC patients received chemotherapy and the ratio between the SBM group and the non-SBM group had no significant difference (*p* = 0.363). 46.0% of all LCC patients received radiotherapy and 23.6% underwent surgery for primary site. Compared with the non-SBM group, the ratio of radiation in patients with SBM was significantly higher (75.5% vs 40.1%), while the rate of surgery was the opposite (4.6% vs 27.2). Baseline characteristics of patients with SBM receiving different treatments are summarized in Additional file 1: Table S1. The result of Kaplan–Meier survival analysis and log-rank test revealed that there was significant differences in the effects of different treatment strategies on OS of LCC with brain metastases (Fig. 3). The surgery combined with chemotherapy and radiotherapy group, in which all patients were N0 stage and had no other site-specific metastases, showed the best median OS of 15 months. The median OS of chemotherapy and radiotherapy group was 6 months, better than that of the chemotherapy alone group or radiotherapy alone group (5 months and 2 months respectively).The no treatment group showed the poorest median OS of just 1 month. Further treatment subgroup analysis was performed both before and after PSM, although some baseline characteristics were not well balanced after PSM due to the small sample size of patients available for selection. The results showed that there was no statistically significant difference in survival between the chemotherapy and radiotherapy group and chemotherapy alone group, as well as between the radiotherapy alone group and the no treatment group (Additional file 1: Tables S2, S3 and Figs. S1, S2).

## Discussion

Due to the low incidence and material changes in WHO Lung Tumors Classification of LCC, few clinical researches have focused on the prognosis of LCC, let alone LCC patients with SBM. To the best of our knowledge, this is the first study to explore the relationship between clinical characteristics and the occurrence as well as prognosis of SBM in LCC. This population-based study indicates that age ≤ 60 and bone metastases are independent risk factors for SBM incidence. Age, T stage, bone metastases and chemotherapy were proved to be independent prognostic factors for OS of LCC patients with SBM. In addition, our study also reveals the prognosis of LCC patients with SBM receiving different treatment schemes.

In the recent past, the histologic subtypes of NSCLC mainly consisted of adenocarcinoma, squamous cell carcinoma and large cell carcinoma (LCC). As the third most common subtype of NSCLC, LCC historically accounted for about 10% of NSCLC, including several subtypes, namely clear cell carcinoma (CCC), lymphoepithelioma-like carcinoma (LELC), LCC with rhabdoid phenotype (LCC-R), basaloid carcinoma (BC), large cell neuroendocrine carcinoma (LCNEC) and the entirely undifferentiated LCC [9]. As the 2015 World Health Organization classification of lung tumors proposed, the diagnosis of large cell carcinoma was restricted only to resected tumors that lack any clear morphologic or immunohistochemical (IHC) differentiation. Using IHC markers, 60–92% of LCC exhibited evidence of either glandular or squamous differentiation, which could be diagnosed as adenocarcinoma or squamous cell carcinoma, respectively. And the above pathological subtypes of LCC were recategorized into other tumor groups, except for only a minor subset of cases remaining fully unclassifiable at both the morphologic and marker expression levels [2, 10]. Therefore, LCC has currently become one of the rarest subtypes of NSCLC, with a prevalence in the low single digits. In our study, the number of patients diagnosed as LCC between 2015 and 2019 dropped by more than half compared with that between 2010 and 2014, which is consistent with advances in pathologic classification of LCC in the same period. Notably, survival analysis showed no difference in OS of LCC patients with SBM between these two periods. Given that brain metastasis is associated with poor prognosis, the potential effect of accurate pathological differentiation between other poorly-differentiated types of NSCLC and LCC would be smaller for these patients.

The incidence of brain metastases is 10–20% in patients with NSCLC at diagnosis, and the lifetime incidence has increased up to 50% as a result of an increase in patients’ survival [11, 12]. Our study based on the latest data found that the incidence of synchronous brain metastases (SBM) in newly diagnosed LCC patients is 15.7%, lower than the previously reported incidence of 17.3% analyzed by Wang et al. from the SEER data (2010–2014) [7], suggesting that the incidence rate of SBM in LCC patients has a downward trend in recent years. According to our result, age and bone metastases are not only independently associated with SBM, but also with shorter OS. However, it is interesting to note that compared with age < 60, age 60–79 is an independent poor prognostic factor for LCC patients with SBM, while age ≥ 80 is not. This paradoxical finding might result from selection bias, as after exclusion of patients with incomplete information, there were only 11 patients aged ≥ 80 with SBM included in the Cox regression analyses. A trend for liver metastases towards statistically significant predictor of SBM also emerged (OR: 1.5; 95% CI 0.96–2.34; *p* = 0.08). High T-stage and chemotherapy were also found to be independently poor prognostic factors for OS. The above findings were consistent with those described in previous studies on other types of lung cancer. Zhu et al. [13] demonstrated that in NSCLC patients, young age (< 65), female, race, higher tumor grade, advanced T-stage or N-stage, liver metastasis, lung metastasis, bone metastasis were independent risk factors for developing SBM, while female, grade, early T stage, married status, no other site-specific metastases, other race, chemotherapy and radiation treatments were associated with significantly better mLCSS. In another retrospective study based on SEER database, Zhou et al. declared that SCLC patients who are black, higher T stage, lung metastases and bone metastases had greater odds of SBM at initial diagnosis, and age ≥ 65, singled, higher T stage, higher N stage, liver metastases and bone metastases were independently related to shorter OS [14].

The reason why the SBM incidence rate of young patients is higher than that of old patients may be complicated. Aging in the central nervous system (CNS) is accompanied by alterations to the brain immune landscape and thus a changed immune contexture stood as a candidate mechanism to affect brain metastatic aggressiveness [15, 16].Wood et al. demonstrated that brain metastases were Two–fourfold higher in young as compared with older mouse hosts in triple-negative or luminal B breast cancer, as aged brains contained fewer resident CNS myeloid cells, which reduced brain metastasis burden [17]. Another possible explanation is that aged patients tend to be more sensitive to symptoms and thus seek medical attention in the early stages. Our study also indicates that patients with extrathoracic metastasis, such as bone liver metastasis and liver metastasis, were more likely to develop synchronous brain metastasis. This seems reasonable because metastasis (from initial primary tumor growth through angiogenesis, intravasation, survival in the bloodstream, extravasation and metastatic growth) is an inefficient process and few released cancer cells complete the entire process [18, 19]. Therefore, the occurrence of hematogenous metastasis reflects the survival of cancer cells within the circulation, which greatly increases the possibility of brain metastasis. This may also shed light on why T stage and N stage failed to present as independent predictors of SBM after multivariable logistic regression analysis.

Up to now, there is still no peculiar guidelines of treatment for LCC with brain metastases. Convincing evidence from clinical research seems difficult to obtain due to the low incidence of this disease. In this study, the mOS of LCC patients with SBM was 4 months, which is much shorter than that of adenocarcinoma (15 months) and nonadenocarcinoma (9 months) with brain metastases [20]. Notably, though the overall population prognosis is poor, our findings revealed that the surgery combined with chemotherapy and radiotherapy group, in which all patients were N0 stage and had no other site-specific metastases, exhibited the best median OS of 15 months. This indicates the importance of primary surgical intervention for highly selected patients. For patients with oligometastatic NSCLC, survival benefit from resection of the primary lung tumor and aggressive local treatments to metastatic sites has been demonstrated in previous studies [21, 22]. And according to the NCCN guidelines of NSCLC, patients who have a single brain metastasis and limited-stage disease in the chest may benefit from aggressive local treatments to both the primary lung cancer and metastatic sites [23]. Our further treatment subgroup analysis suggests that chemotherapy provides a reliable survival benefit for LCC with brain metastases, whereas radiotherapy provided a feeble survival benefit. This is in accordance with previous studies suggesting an improved intracranial response rate with the combination of chemotherapy and radiotherapy versus chemotherapy alone but no OS differences [24, 25].

There are several limitations in our study. First, due to the retrospective nature of this study and the processing of the data, selection bias could therefore not be avoided as mentioned above. Second, the SEER database did not record some key factors, such as performance status, smoking status, the number of BM, RPA classification, driver mutations and the details of treatment regimens. Loss of these information might affect our research results to some extent. Finally, although PSM was used to reduce selection bias in the treatment subgroup analysis, limited number of patients made it difficult to avoid bias in all confounding factors. Despite these limitations, the present study included the greatest number of LCC patients with synchronous brain metastases and provided relatively reliable clinical values.

## Conclusion

Based on our findings, it is evident that LCC patients with age < 60 or bone metastases were more likely to have SBM at initial diagnosis. Age, T stage, bone metastases and chemotherapy were identified to be independent prognostic factors for OS of LCC patients with SBM. Notably, though the overall population prognosis was poor, highly selected patients might achieve the best survival benefit from surgery combined with chemotherapy and radiotherapy.

## Supplementary Information

**Additional file 1**.** Table S1**. Baseline characteristics of patients with SBM receiving different treatments.** Table S2**. Baseline characteristics of patients with SBM receiving chemotherapy only and chemotherapy plus radiotherapy before and after PSM.** Table S3**. Baseline characteristics of patients with SBM receiving radiotherapy only and no treatment before and after PSM.** Figure S1**. Kaplan–Meier curves of LCC patients with SBM receiving chemotherapy and radiotherapy vs radiotherapy before and after 1:2 PSM.** Figure S2**. Kaplan–Meier curves of LCC patients with SBM receiving radiotherapy vs no treatment before and after 1:1 PSM.

## Data Availability

The datasets used and/or analysed during the current study are available from the corresponding author on reasonable request.

## Abbreviations

LCC: Large cell carcinoma
SBM: Synchronous brain metastases
SEER: Surveillance, epidemiology, and end results database
PSM: Propensity score-matching
OS: Overall survival
NSCLC: Non-small cell lung cancer
NOS: Not otherwise specified
AJCC: American Joint Committee on Cancer
LCSS: Lung cancer-specific survival
