# Supplementary material for: Factors for incidence risk and prognosis of synchronous brain metastases in pulmonary large cell carcinoma patients: a population-based study
Source: BMC Pulm Med. 2023 Jan 12;23:12. doi: 10.1186/s12890-023-02312-y (PMC9835350; doi:10.1186/s12890-023-02312-y)
Supplement: Supplementary file 1 — Additional file 1. Table S1. Baseline characteristics of patients with SBM receiving different treatments. Table S2. Baseline characteristics of patients with SBM receiving chemotherapy only and chemotherapy plus radiotherapy before and after PSM. Table S3. Baseline characteristics of patients with SBM receiving radiotherapy only and no treatment before and after PSM. Figure S1. Kaplan–Meier curves of LCC patients with SBM receiving chemotherapy and radiotherapy vs radiotherapy before and after 1:2 PSM. Figure S2. Kaplan–Meier curves of LCC patients with SBM receiving radiotherapy vs no treatment before and after 1:1 PSM. [file 12890_2023_2312_MOESM1_ESM.docx]

| **Table S1 Baseline Patient Characteristics** | | | | | | |
| --- | --- | --- | --- | --- | --- | --- |
|  | **Surgery** |  |  |  |  |  |
|  | **Chemotherapy** | **Chemotherapy** |  |  |  |  |
|  | **Radiotherapy** | **Radiotherapy** | **Chemotherapy** | **Radiotherapy** | **No Treatment** |  |
| **Characteristics** | **N = 6** | **N = 91** | **N = 13** | **N = 63** | **N = 39** | **P Value** |
| Year of diagnosis |  |  |  |  |  | 0.906 |
| 2010-2014 | 4 ( 66.7) | 69 (75.8) | 10 (76.9) | 46 (73.0) | 27 (69.2) |  |
| 2015-2019 | 2 ( 33.3) | 22 (24.2) | 3 (23.1) | 17 (27.0) | 12 (30.8) |  |
| Age |  |  |  |  |  | 0.204 |
| ＜60 | 5 ( 83.3) | 40 (44.0) | 4 (30.8) | 20 (31.7) | 11 (28.2) |  |
| 60-79 | 1 ( 16.7) | 48 (52.7) | 8 (61.5) | 39 (61.9) | 25 (64.1) |  |
| ≥80 | 0 ( 0.0) | 3 ( 3.3) | 1 ( 7.7) | 4 ( 6.3) | 3 ( 7.7) |  |
| Sex |  |  |  |  |  | 0.250 |
| Male | 6 (100.0) | 48 (52.7) | 7 (53.8) | 37 (58.7) | 22 (56.4) |  |
| Female | 0 ( 0.0) | 43 (47.3) | 6 (46.2) | 26 (41.3) | 17 (43.6) |  |
| Race |  |  |  |  |  | 0.954 |
| White | 5 ( 83.3) | 72 (79.1) | 12 (92.3) | 52 (82.5) | 34 (87.2) |  |
| Black | 1 ( 16.7) | 13 (14.3) | 1 ( 7.7) | 9 (14.3) | 4 (10.3) |  |
| Other | 0 ( 0.0) | 6 ( 6.6) | 0 ( 0.0) | 2 ( 3.2) | 1 ( 2.6) |  |
| Marital status |  |  |  |  |  | 0.669 |
| Married | 4 ( 66.7) | 50 (54.9) | 6 (46.2) | 28 (44.4) | 19 (48.7) |  |
| Single | 2 ( 33.3) | 41 (45.1) | 7 (53.8) | 35 (55.6) | 20 (51.3) |  |
| Median household income |  |  |  |  |  | 0.419 |
| <$55,000 | 3 ( 50.0) | 35 (38.5) | 4 (30.8) | 28 (44.4) | 9 (23.1) |  |
| $55,000 - $74,999 | 1 ( 16.7) | 43 (47.3) | 6 (46.2) | 26 (41.3) | 23 (59.0) |  |
| $75,000+ | 2 ( 33.3) | 13 (14.3) | 3 (23.1) | 9 (14.3) | 7 (17.9) |  |
| Primary Site |  |  |  |  |  | 0.378 |
| Main bronchus | 0 ( 0.0) | 3 ( 3.3) | 0 ( 0.0) | 6 ( 9.5) | 0 ( 0.0) |  |
| Upper lobe | 6 (100.0) | 54 (59.3) | 9 (69.2) | 35 (55.6) | 21 (53.8) |  |
| Middle lobe | 0 ( 0.0) | 1 ( 1.1) | 1 ( 7.7) | 2 ( 3.2) | 1 ( 2.6) |  |
| Lower lobe | 0 ( 0.0) | 24 (26.4) | 3 (23.1) | 12 (19.0) | 12 (30.8) |  |
| Unspecific | 0 ( 0.0) | 9 ( 9.9) | 0 ( 0.0) | 8 (12.7) | 5 (12.8) |  |
| Laterality |  |  |  |  |  | 0.923 |
| Left | 3 ( 50.0) | 42 (46.2) | 7 (53.8) | 25 (39.7) | 18 (46.2) |  |
| Right | 3 ( 50.0) | 48 (52.7) | 6 (46.2) | 36 (57.1) | 21 (53.8) |  |
| Bilateral | 0 ( 0.0) | 1 ( 1.1) | 0 ( 0.0) | 2 ( 3.2) | 0 ( 0.0) |  |
| Grade |  |  |  |  |  | 0.027 |
| III | 3 ( 50.0) | 23 (25.3) | 9 (69.2) | 18 (28.6) | 14 (35.9) |  |
| IV | 3 ( 50.0) | 23 (25.3) | 2 (15.4) | 19 (30.2) | 7 (17.9) |  |
| Unknown | 0 ( 0.0) | 45 (49.5) | 2 (15.4) | 26 (41.3) | 18 (46.2) |  |
| T stage |  |  |  |  |  | 0.529 |
| T1 | 3 ( 50.0) | 18 (19.8) | 2 (15.4) | 10 (15.9) | 9 (23.1) |  |
| T2 | 3 ( 50.0) | 27 (29.7) | 4 (30.8) | 14 (22.2) | 7 (17.9) |  |
| T3 | 0 ( 0.0) | 35 (38.5) | 4 (30.8) | 32 (50.8) | 17 (43.6) |  |
| T4 | 0 ( 0.0) | 1 ( 1.1) | 0 ( 0.0) | 1 ( 1.6) | 0 ( 0.0) |  |
| T0 | 0 ( 0.0) | 10 (11.0) | 3 (23.1) | 6 ( 9.5) | 6 (15.4) |  |
| N stage |  |  |  |  |  | 0.006 |
| N0 | 6 (100.0) | 22 (24.2) | 2 (15.4) | 14 (22.2) | 9 (23.1) |  |
| N1 | 0 ( 0.0) | 11 (12.1) | 0 ( 0.0) | 4 ( 6.3) | 6 (15.4) |  |
| N2 | 0 ( 0.0) | 39 (42.9) | 7 (53.8) | 34 (54.0) | 21 (53.8) |  |
| N3 | 0 ( 0.0) | 19 (20.9) | 4 (30.8) | 11 (17.5) | 3 ( 7.7) |  |
| Bone metastases |  |  |  |  |  | 0.151 |
| No | 6 (100.0) | 59 (64.8) | 6 (46.2) | 40 (63.5) | 29 (74.4) |  |
| Yes | 0 ( 0.0) | 32 (35.2) | 7 (53.8) | 23 (36.5) | 10 (25.6) |  |
| Liver metastases |  |  |  |  |  | 0.550 |
| No | 6 (100.0) | 77 (84.6) | 10 (76.9) | 49 (77.8) | 30 (76.9) |  |
| Yes | 0 ( 0.0) | 14 (15.4) | 3 (23.1) | 14 (22.2) | 9 (23.1) |  |
| Lung metastases |  |  |  |  |  | 0.861 |
| No | 6 (100.0) | 73 (80.2) | 10 (76.9) | 50 (79.4) | 30 (76.9) |  |
| Yes | 0 ( 0.0) | 18 (19.8) | 3 (23.1) | 13 (20.6) | 9 (23.1) |  |
| Abbreviations: T, Tumor; N, Node. | | | | | | |

| **Table S2 Baseline Patient Characteristics** | | | | | | |
| --- | --- | --- | --- | --- | --- | --- |
|  | **Before PSM** | | | **After PSM (1:2)** | | |
|  |  | **Chemotherapy** |  |  | **Chemotherapy** |  |
|  | **Chemotherapy** | **Radiotherapy** |  | **Chemotherapy** | **Radiotherapy** |  |
| **Characteristics** | **N = 13** | **N = 91** | **P Value** | **N = 13** | **N = 26** | **P Value** |
| Year of diagnosis |  |  | 0.931 |  |  | 1 |
| 2010-2014 | 10 (76.92) | 69 (75.82) |  | 10 (76.92) | 20 (76.92) |  |
| 2015-2019 | 3 (23.08) | 22 (24.18) |  | 3 (23.08) | 6 (23.08) |  |
| Age |  |  | 0.5482 |  |  | 0.8737 |
| ＜60 | 4 (30.77) | 40 (43.96) |  | 4 (30.77) | 8 (30.77) |  |
| 60-79 | 8 (61.54) | 48 (52.75) |  | 8 (61.54) | 17 (65.38) |  |
| ≥80 | 1 (7.69) | 3 (3.30) |  | 1 (7.69) | 1 (3.85) |  |
| Sex |  |  | 0.941 |  |  | 0.9084 |
| Male | 7 (53.85) | 48 (52.75) |  | 7 (53.85) | 16 (61.54) |  |
| Female | 6 (46.15) | 43 (47.25) |  | 6 (46.15) | 10 (38.46) |  |
| Race |  |  | 0.4797 |  |  | 0.709 |
| White | 12 (92.31) | 72 (79.12) |  | 12 (92.31) | 23 (88.46) |  |
| Black | 1 (7.69) | 13 (14.29) |  | 1 (7.69) | 3 (11.54) |  |
| Other | 0 (0.00) | 6 (6.59) |  | 0 (0.00) | 0 (0.00) |  |
| Marital status |  |  | 0.7662 |  |  | 1 |
| Married | 6 (46.15) | 50 (54.95) |  | 6 (46.15) | 12 (46.15) |  |
| Single | 7 (53.85) | 41 (45.05) |  | 7 (53.85) | 14 (53.85) |  |
| Median household income |  |  | 0.686 |  |  | 0.6997 |
| <$55,000 | 4 (30.77) | 35 (38.46) |  | 4 (30.77) | 5 (19.23) |  |
| $55,000 - $74,999 | 6 (46.15) | 43 (47.25) |  | 6 (46.15) | 15 (57.69) |  |
| $75,000+ | 3 (23.08) | 13 (14.29) |  | 3 (23.08) | 6 (23.08) |  |
| Primary Site |  |  | 0.3405 |  |  | 0.3951 |
| Main bronchus | 0 (0.00) | 3 (3.30) |  | 0 (0.00) | 1 (3.85) |  |
| Upper lobe | 9 (69.23) | 54 (59.34) |  | 9 (69.23) | 17 (65.38) |  |
| Middle lobe | 1 (7.69) | 1 (1.10) |  | 1 (7.69) | 0 (0.00) |  |
| Lower lobe | 3 (23.08) | 24 (26.37) |  | 3 (23.08) | 5 (19.23) |  |
| Unspecific | 0 (0.00) | 9 (9.89) |  | 0 (0.00) | 3 (11.54) |  |
| Laterality |  |  | 0.8266 |  |  | 0.7292 |
| Left | 7 (53.85) | 42 (46.15) |  | 7 (53.85) | 12 (46.15) |  |
| Right | 6 (46.15) | 48 (52.75) |  | 6 (46.15) | 13 (50.00) |  |
| Bilateral | 0 (0.00) | 1 (1.10) |  | 0 (0.00) | 1 (3.85) |  |
| Grade |  |  | 0.0052 |  |  | 0.7753 |
| III | 9 (69.23) | 23 (25.27) |  | 9 (69.23) | 15 (57.69) |  |
| IV | 2 (15.38) | 23 (25.27) |  | 2 (15.38) | 5 (19.23) |  |
| Unknown | 2 (15.38) | 45 (49.45) |  | 2 (15.38) | 6 (23.08) |  |
| T stage |  |  | 0.7778 |  |  | 0.6174 |
| T1 | 3 (23.08) | 10 (10.99) |  | 3 (23.08) | 3 (11.54) |  |
| T2 | 2 (15.38) | 18 (19.78) |  | 2 (15.38) | 8 (30.77) |  |
| T3 | 4 (30.77) | 27 (29.67) |  | 4 (30.77) | 9 (34.62) |  |
| T4 | 4 (30.77) | 35 (38.46) |  | 4 (30.77) | 6 (23.08) |  |
| T0 | 0 (0.00) | 1 (1.10) |  | 0 (0.00) | 0 (0.00) |  |
| N stage |  |  | 0.4291 |  |  | 0.6494 |
| N0 | 2 (15.38) | 22 (24.18) |  | 2 (15.38) | 6 (23.08) |  |
| N1 | 0 (0.00) | 11 (12.09) |  | 0 (0.00) | 0 (0.00) |  |
| N2 | 7 (53.85) | 39 (42.86) |  | 7 (53.85) | 10 (38.46) |  |
| N3 | 4 (30.77) | 19 (20.88) |  | 4 (30.77) | 10 (38.46) |  |
| Bone metastases |  |  | 0.3196 |  |  | 1 |
| No | 6 (46.15) | 59 (64.84) |  | 6 (46.15) | 12 (46.15) |  |
| Yes | 7 (53.85) | 32 (35.16) |  | 7 (53.85) | 14 (53.85) |  |
| Liver metastases |  |  | 0.7637 |  |  | 0.779 |
| No | 10 (76.92) | 77 (84.62) |  | 10 (76.92) | 21 (80.77) |  |
| Yes | 3 (23.08) | 14 (15.38) |  | 3 (23.08) | 5 (19.23) |  |
| Lung metastases |  |  | 0.782 |  |  | 0.795 |
| No | 10 (76.92) | 73 (80.22) |  | 10 (76.92) | 19 (73.08) |  |
| Yes | 3 (23.08) | 18 (19.78) |  | 3 (23.08) | 7 (26.92) |  |
| Abbreviations: PSM, Propensity-score matching; T, Tumor; N, Node. | | | | | | |

| **Table S3 Baseline Patient Characteristics** | | | | | | |
| --- | --- | --- | --- | --- | --- | --- |
|  | **Before PSM** | | | **After PSM (1:1)** | | |
|  | **Radiotherapy** | **No Treatment** |  | **Radiotherapy** | **No Treatment** |  |
| **Characteristics** | **N = 13** | **N = 91** | **P Value** | **N = 13** | **N = 26** | **P Value** |
| Year of diagnosis |  |  | 0.8525 |  |  | 0.808 |
| 2010-2014 | 27 (69.23) | 46 (73.02) |  | 27 (69.23) | 26 (66.67) |  |
| 2015-2019 | 12 (30.77) | 17 (26.98) |  | 12 (30.77) | 13 (33.33) |  |
| Age |  |  | 0.9134 |  |  | 0.8825 |
| ＜60 | 11 (28.21) | 20 (31.75) |  | 11 (28.21) | 13 (33.33) |  |
| 60-79 | 25 (64.10) | 39 (61.90) |  | 25 (64.10) | 23 (58.97) |  |
| ≥80 | 3 (7.69) | 4 (6.35) |  | 3 (7.69) | 3 (7.69) |  |
| Sex |  |  | 0.9806 |  |  | 0.8179 |
| Male | 22 (56.41) | 37 (58.73) |  | 22 (56.41) | 24 (61.54) |  |
| Female | 17 (43.59) | 26 (41.27) |  | 17 (43.59) | 15 (38.46) |  |
| Race |  |  | 0.8192 |  |  | 0.9243 |
| White | 34 (87.18) | 52 (82.54) |  | 34 (87.18) | 35 (89.74) |  |
| Black | 4 (10.26) | 9 (14.29) |  | 4 (10.26) | 3 (7.69) |  |
| Other | 1 (2.56) | 2 (3.17) |  | 1 (2.56) | 1 (2.56) |  |
| Marital status |  |  | 0.8287 |  |  | 0.821 |
| Married | 19 (48.72) | 28 (44.44) |  | 19 (48.72) | 18 (46.15) |  |
| Single | 20 (51.28) | 35 (55.56) |  | 20 (51.28) | 21 (53.85) |  |
| Median household income |  |  | 0.0903 |  |  | 0.3581 |
| <$55,000 | 9 (23.08) | 28 (44.44) |  | 9 (23.08) | 14 (35.90) |  |
| $55,000 - $74,999 | 23 (58.97) | 26 (41.27) |  | 23 (58.97) | 17 (43.59) |  |
| $75,000+ | 7 (17.95) | 9 (14.29) |  | 7 (17.95) | 8 (20.51) |  |
| Primary Site |  |  | 0.2708 |  |  | 0.5984 |
| Main bronchus | 0 (0.00) | 6 (9.52) |  | 0 (0.00) | 2 (5.13) |  |
| Upper lobe | 21 (53.85) | 35 (55.56) |  | 21 (53.85) | 21 (53.85) |  |
| Middle lobe | 1 (2.56) | 2 (3.17) |  | 1 (2.56) | 2 (5.13) |  |
| Lower lobe | 12 (30.77) | 12 (19.05) |  | 12 (30.77) | 9 (23.08) |  |
| Unspecific | 5 (12.82) | 8 (12.70) |  | 5 (12.82) | 5 (12.82) |  |
| Laterality |  |  | 0.4667 |  |  | 0.5979 |
| Left | 18 (46.15) | 25 (39.68) |  | 18 (46.15) | 17 (43.59) |  |
| Right | 21 (53.85) | 36 (57.14) |  | 21 (53.85) | 21 (53.85) |  |
| Bilateral | 0 (0.00) | 2 (3.17) |  | 0 (0.00) | 1 (2.56) |  |
| Grade |  |  | 0.3764 |  |  | 0.9685 |
| III | 14 (35.90) | 18 (28.57) |  | 14 (35.90) | 13 (33.33) |  |
| IV | 7 (17.95) | 19 (30.16) |  | 7 (17.95) | 7 (17.95) |  |
| Unknown | 18 (46.15) | 26 (41.27) |  | 18 (46.15) | 19 (48.72) |  |
| T stage |  |  | 0.6505 |  |  | 0.5933 |
| T1 | 6 (15.38) | 6 (9.52) |  | 6 (15.38) | 5 (12.82) |  |
| T2 | 9 (23.08) | 10 (15.87) |  | 9 (23.08) | 5 (12.82) |  |
| T3 | 7 (17.95) | 14 (22.22) |  | 7 (17.95) | 10 (25.64) |  |
| T4 | 17 (43.59) | 32 (50.79) |  | 17 (43.59) | 18 (46.15) |  |
| T0 | 0 (0.00) | 1 (1.59) |  | 0 (0.00) | 1 (2.56) |  |
| N stage |  |  | 0.2972 |  |  | 0.7051 |
| N0 | 9 (23.08) | 14 (22.22) |  | 9 (23.08) | 8 (20.51) |  |
| N1 | 6 (15.38) | 4 (6.35) |  | 6 (15.38) | 3 (7.69) |  |
| N2 | 21 (53.85) | 34 (53.97) |  | 21 (53.85) | 24 (61.54) |  |
| N3 | 3 (7.69) | 11 (17.46) |  | 3 (7.69) | 4 (10.26) |  |
| Bone metastases |  |  | 0.3564 |  |  | 1 |
| No | 29 (74.36) | 40 (63.49) |  | 29 (74.36) | 29 (74.36) |  |
| Yes | 10 (25.64) | 23 (36.51) |  | 10 (25.64) | 10 (25.64) |  |
| Liver metastases |  |  | 0.92 |  |  | 1 |
| No | 30 (76.92) | 49 (77.78) |  | 30 (76.92) | 30 (76.92) |  |
| Yes | 9 (23.08) | 14 (22.22) |  | 9 (23.08) | 9 (23.08) |  |
| Lung metastases |  |  | 0.9651 |  |  | 0.792 |
| No | 30 (76.92) | 50 (79.37) |  | 30 (76.92) | 29 (74.36) |  |
| Yes | 9 (23.08) | 13 (20.63) |  | 9 (23.08) | 10 (25.64) |  |
| Abbreviations: PSM, Propensity-score matching; T, Tumor; N, Node. | | | | | | |


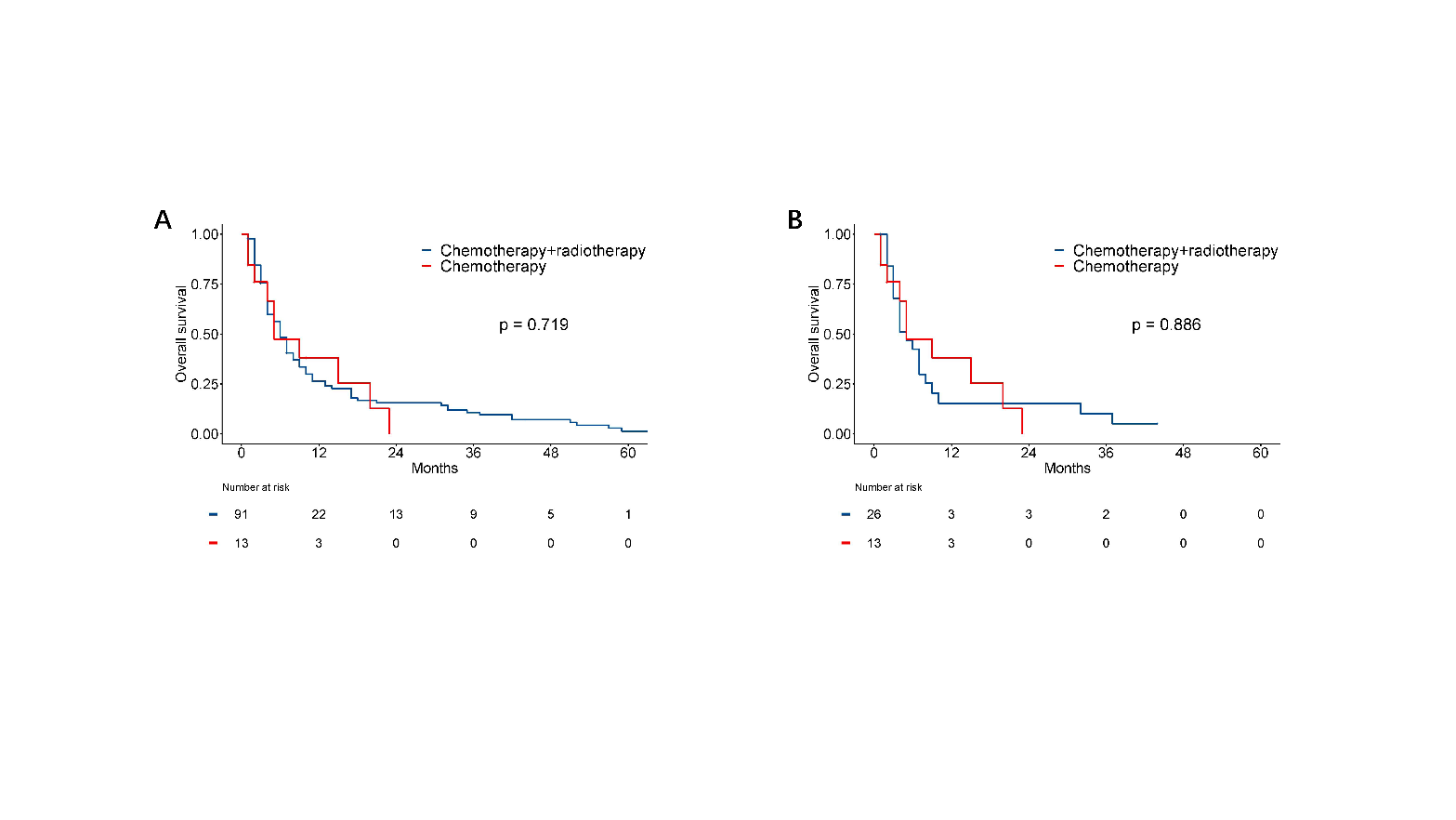


**FIGURE S1** Kaplan–Meier curves of LCC patients with SBM receiving chemotherapy and radiotherapy vs radiotherapy before PSM (A) and after 1:2 PSM (B). LCC, large cell carcinoma; SBM, synchronous brain metastases; PSM, propensity score matching.


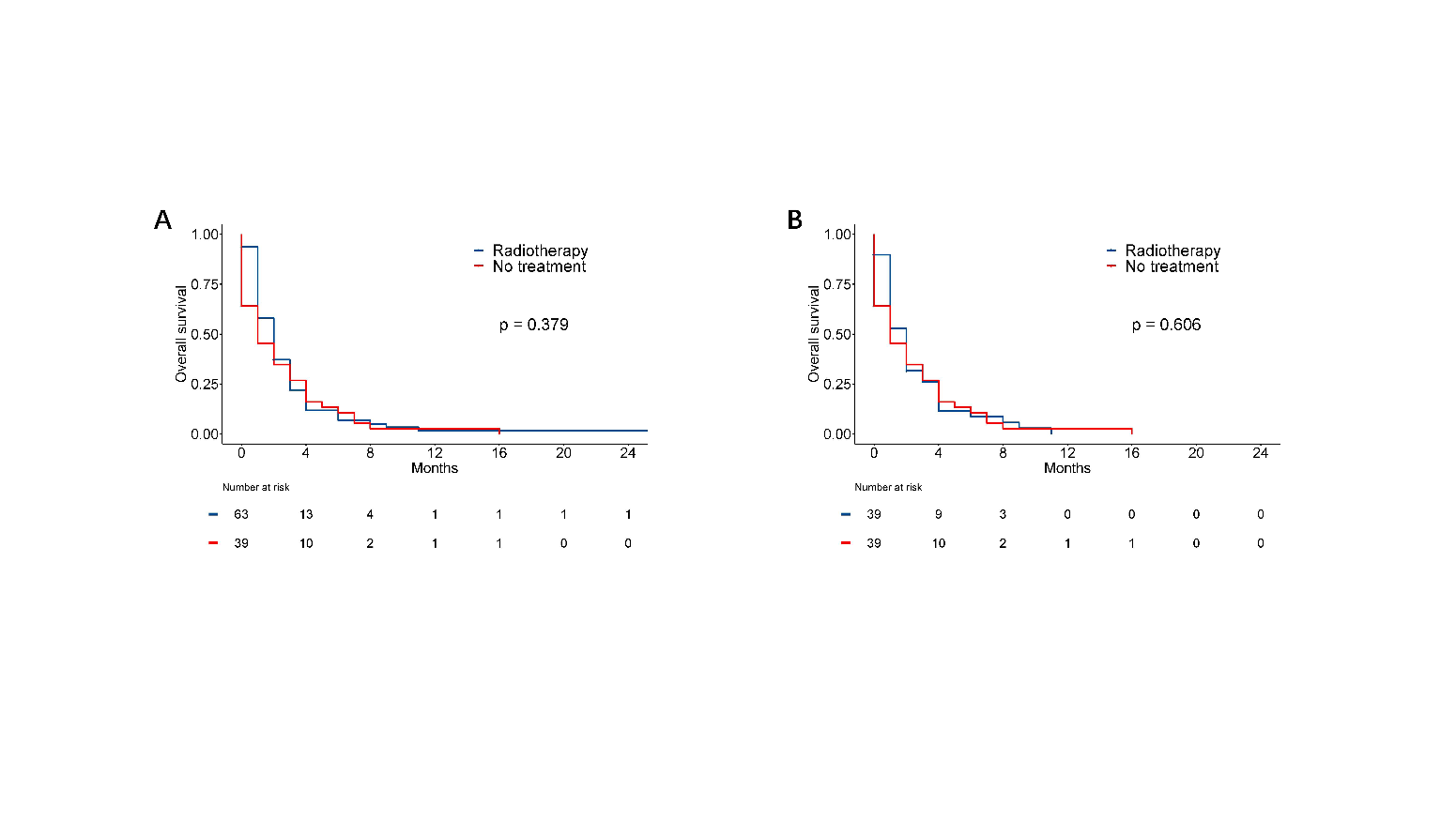


**FIGURE S2** Kaplan–Meier curves of LCC patients with SBM receiving radiotherapy vs No treatment before PSM (A) and after 1:1 PSM (B). LCC, large cell carcinoma; SBM, synchronous brain metastases; PSM, propensity score matching.
